# Evaluation of the Efficacy and Safety of PARP Inhibitors in Advanced-Stage Epithelial Ovarian Cancer

**DOI:** 10.3389/fonc.2020.00954

**Published:** 2020-07-03

**Authors:** Yifan Jiang, Juan Zhao, Li Zhang, Sijuan Tian, Ting Yang, Li Wang, Minyi Zhao, Qing Yang, Yaohui Wang, Xiaofeng Yang

**Affiliations:** Department of Gynecology and Obstetrics, The First Affiliated Hospital of Xi'an Jiaotong University, Xi'an, China

**Keywords:** PARP inhibitors, maintenance treatment, epithelial ovarian cancer, rucaparib, olaparib, niraparib, veliparib, meta-analysis

## Abstract

**Purpose:** PARP inhibitors are a novel targeted anti-cancer drug and a large number of clinical studies on PARP inhibitors have been accomplished. This updated meta-analysis was conducted to evaluate the efficacy and safety of PARP inhibitors in advanced-stage epithelial ovarian cancer.

**Methods:** Medline (PubMed), Embase, Cochrane Central Register of Controlled Trials, Web of Science, and Scopus were searched to identify the eligible trials up to April 2020. ClinicalTrials.gov was also screened for additional unpublished trials. Data extraction and risk of bias assessment were performed by two independent investigators, respectively. The hazard ratios (HRs) and its 95% confidence intervals (CI) for time-to-event data of progression-free survival (PFS) and overall survival (OS), and the risk ratios (RRs) with 95% CI for dichotomous data of overall response rate (ORR) and occurrence of adverse events (AEs) were calculated by Review Manager 5.3 and Stata 12.0 software.

**Results:** A total of 12 trials with 5,347 patients were included in this meta-analysis. Compared with the control group, PARP inhibitors significantly improved PFS (HR, 0.51; 95% CI, 0.40–0.65; *P* < 0.00001) and ORR (RR, 1.26; 95% CI, 1.11–1.43; *P* = 0.0003). Specifically, PFS was improved regardless of *BRCA* genes mutations and homologous-recombination status. However, no difference was observed in OS between the PARP inhibitors group and the control group (95% CI, 0.73–1.01; *P* = 0.06). PARP inhibitors were associated with a statistically significant higher risk of hematologic events and different PARP inhibitors had different toxicities profiles.

**Conclusion:** PARP inhibitors are an effective and well-tolerated treatment for patients with advanced-stage epithelial ovarian cancer.

## Introduction

Epithelial ovarian cancer (EOC) is the most lethal gynecological malignancy with a 5-years survival rate of only 29% for the advanced stage (1). The current standard treatment of EOC is cytoreductive surgery combined with platinum-based chemotherapy. Unfortunately, despite the initial response to chemotherapy, up to 80% of the patients with advanced ovarian cancer experience relapse with a median PFS of 12–18 months and even become resistant to subsequent therapy (2). In this scenario, the long-term survival for late-stage patients with OC has not increased significantly in the past 25 years (3). It remains urgent to develop more therapies to improve long-term disease control of EOC.

Poly (ADP-ribose) polymerase (PARP) inhibitors are the novel targeted therapies that have a big impact on the clinical management of EOC (4). PARP is an attractive antitumor target with a catalytic site that transfers an ADP-ribose group on specific acceptor proteins using NAD+ as cofactor. This post-translational protein modification is named PARylation and the acceptor proteins include a variety of histones and PARP itself (auto-PARylation), which allows PARPs to involve in different cellular activities. During the DNA damage response, PARP1 detects the site of single-strand breaks (SSB) and docks such DNA repair proteins as topoisomerase, DNA ligase III, and scaffolding proteins by PARylation (5). PARP2 is also known to be involved in the SSB repair pathway (6) and all clinical PARP inhibitors target both PARP1 and PARP2. PARP inhibitors prevent SSB repair and result in the formation of DNA double-stranded breaks (DSB) which cannot be accurately repaired in tumors with homologous-recombination deficiency (HRD), such as tumors with deleterious mutations in *BRCA1/2*. This strategy is defined as synthetic lethality that the cooperation between pharmacological toxicity of PARP inhibitors and gene defects in homologous-recombination repair pathway leads to cell death eventually (7, 8). However, homologous recombination repair pathway involves not only *BRCA* but also other genes like *ATM* or *PALB2* (9), which partially explain why OC patients with wild-type *BRCA* can also benefit from PARP inhibitors treatment. The estimated prevalence of a germline or somatic *BRCA1/2* mutation in ovarian cancer is about 20%, and genomic defects involved in homologous-recombination is up to 30% (10, 11). Actually, the mechanism of action of PARP inhibitors is not only related to catalytic inhibition through competitively interacting with NAD+ binding site of PARP1/2, but also trapping the PARP-DNA complexes through a conformational change (12). Interestingly, it is the trapping potent other than the ability to inhibit PARylation that determines therapeutic effect of PARP inhibitors (13). At present, olaparib (14, 15), rucaparib (16, 17), and niraparib (18) have been approved as maintenance treatment in patients with advanced ovarian cancer and a large number of clinical trials including other PARP inhibitors like veliparib were undergone. Patients can be included regardless of *BRCA* mutation status or homologous recombination status in several trials. Now that the data of several randomized controlled trials (RCTs) have become available, this updated meta-analysis was performed to investigate the efficacy and safety of PARP inhibitors.

## Materials and Methods

This meta-analysis was a quantitative synthesis of RCTs evaluating the efficacy and safety of PARP inhibitors compared with a control drug (chemotherapy or placebo) in patients with advanced-stage epithelial ovarian cancer. This meta-analysis was performed in adherence to the Preferred Reporting Items for Systematic Reviews and Meta-Analyses (PRISMA) statement (19).

### Data Sources and Search Strategy

To identify the eligible trials up to April 2020, five databases—Medline (PubMed), Embase, Cochrane Central Register of Controlled Trials, Web of Science, and Scopus—were searched systematically and comprehensively. The search terms are as following: (“Ovarian Neoplasms”[Mesh] OR ovarian cancer OR ovarian tumor OR ovarian carcinoma OR epithelial ovarian cancer) and (“Poly(ADP-ribose) Polymerase Inhibitors”[Mesh] OR PARP inhibitors OR “rucaparib” [Supplementary Concept] OR AG014699 OR “olaparib” [Supplementary Concept] OR Lynparza OR AZD2281 OR “niraparib” [Supplementary Concept] OR MK4827 OR “veliparib” [Supplementary Concept] OR ABT888) and maintenance treatment and (“Randomized Controlled Trial” [Publication Type] OR RCT). Additionally, the ClinicalTrials.gov was also screened to obtain more information on the registered RCTs. Finally, all references of included articles were reviewed manually for more potential trials.

### Inclusion and Exclusion Criteria

Included trials were required to meet the following criteria: (1) RCTs comparing PARP inhibitors or PARP inhibitors plus chemotherapy in the intervention arm with placebo or chemotherapy or chemotherapy plus placebo in the control arm; (2) women aged 18 years or older with histologically or cytologically diagnosed epithelial ovarian cancer, primary peritoneal cancer, or fallopian tube cancer; (3) sufficient data to assess efficacy outcomes (PFS, OS, and ORR) and safety outcomes.

Exclusion criteria were mainly as follows: (1) reviews, meta-analysis, commentaries, or conference abstracts; (2) non-RCTs and non-human clinical trials like *in vitro* or animal experiments; (3) trials with incomplete data; (4) participants complicated with other malignant tumors, or severe circulatory diseases or abnormal liver and kidney function.

### Data Extraction

Data extraction was performed by two investigators independently and discrepancies were resolved by consensus or a third reviewer. For each eligible trial, the collected information included the trial name, the first author, publication or presentation year, trial design, number of patients, type of PARP inhibitors, type of control group, previous treatment, *BRCA* mutation status, median PFS, HRs and 95% CI for PFS and OS, ORR and occurrence of AEs in each arm.

### Risk of Bias Assessment

Two reviewers evaluated the quality of the eligible trials independently and disagreements were resolved through discussion. The assessment was based on the Cochrane risk-of-bias tool including sequence generation (selection bias), allocation sequence concealment (selection bias), blinding of participants and personnel (performance bias), blinding of outcome assessment (detection bias), incomplete outcome data (attrition bias), selective outcome reporting (reporting bias), and other potential sources of bias. The judgment for each entry involved assessing the risk of bias as “low risk,” “high risk,” or as “unclear risk” and the total result was presented as percentages in a figure (20).

### Statistical Analyses

The pooled HRs with 95% CI were calculated by the generic inverse of variance method and the pooled RRs with 95% CI were calculated by the Mantel-Haenszel method. The comparison was considered significant when *P* < 0.05 which was calculated by a *Z*-test. Both a random-effect model and a fixed-effect model were used to calculate the pooled HRs and RRs, which was determined by the heterogeneity.

Heterogeneity was evaluated by Chi-squared tests and *I*^2^ statistics. Heterogeneity was considered significant if *P* < 0.1 and *I*^2^ > 50% and a random-effect model was used. Otherwise, a fixed-effect model was used. Subgroup analyses were performed to explore the potential heterogeneity factors.

To evaluate the stability of the overall results, the sensitivity analysis was performed by omitting individual trials one by one. Finally, Begg's funnel plot and Egger's test were used to detect the publication bias. All *P-*values were two-sided and all statistical analyses were performed using Review Manager 5.3 and Stata 12.0 software.

## Results

### Trials Selection

**Figure 1:**
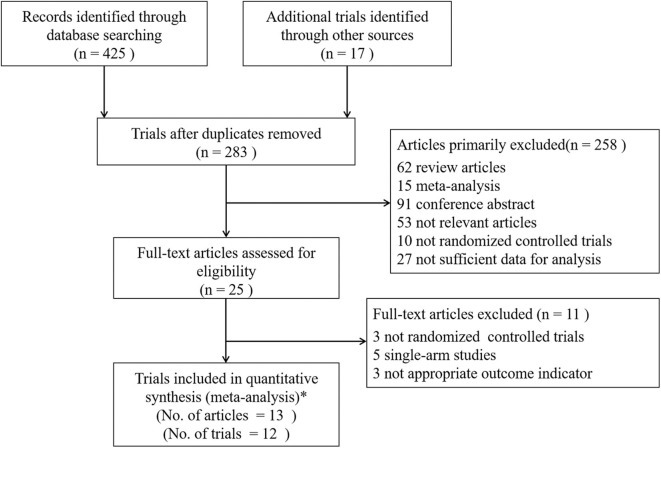
Flow Diagram of Trials Selection. *Finally, 13 articles of 11 trials and 1 additional unpublished trial (SOLO3) with completed results were included. Study 19 had three different articles which were published by Ledermann et al. (14, 21, 22). Analysis of outcomes according to *BRCA* status was published in 2014 and the updated overall survival analysis was published in 2016 at 77% data maturity.

After searching the electronic databases and clinical trial registration website systematically, a total of 425 published articles and 17 trials with results were initially retrieved (Figure 1). One hundred and fifty-nine duplicates were found by Endnote software and removed; 258 articles were excluded according to the criteria after reviewing the titles and abstracts. Further screening the remaining 25 full-text articles made 11 trials omitted, including three not randomized controlled trials, five single-arm studies, and three trials with inappropriate outcome indicators. Ultimately, 13 published articles in peer-reviewed journals from 11 trials and one additional unpublished trial (SOLO3) with completed results from the clinical trial registration website were included in the quantitative synthesis. Notably, three articles from Study 19 which were published by Ledermann et al. (14, 21, 22) were all considered eligible in the final analysis. The paper in 2014 was a preplanned retrospective analysis of outcomes by *BRCA* status of Study 19 as the data on *BRCA* mutational status were not yet available in 2012. And the paper in 2016 referred to an updated overall survival analysis of Study 19 at a higher data maturity of 77% which was only 38% in 2012 and 58% in 2014, respectively. However, the results of these three articles were not adopted in the same analysis item at the same time to avoid repetition.

### Trial Characteristics

**Figure 2:**
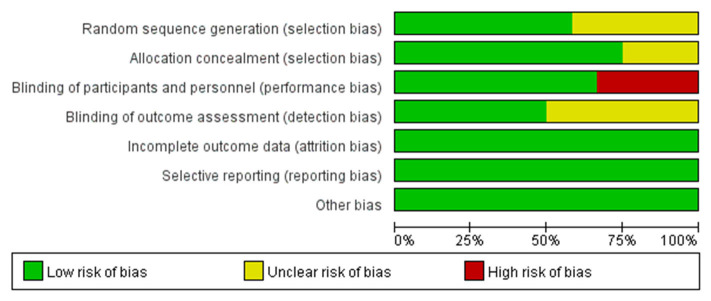
Risk of bias graph.

A total of 5,347 patients from 4 phase II trials and 8 phase III trials were eligible for the final analysis, including 4 open-label trials, of which one evaluated rucaparib, two veliparib, two niraparib, and seven olaparib. The characteristics of the included trials were shown in Table 1. VELIA was a 3-arm trial with patients receiving carboplatin and paclitaxel (PC) plus placebo followed by placebo maintenance (control), PC plus veliparib followed by placebo maintenance (veliparib combination only), or PC plus veliparib followed by veliparib maintenance (veliparib throughout). The veliparib-combination-only group investigated veliparib plus first-line induction chemotherapy with PC as the primary treatment for newly diagnosed advanced ovarian cancer. The veliparib-throughout group of VELIA, as well as PRIMA, SOLO1, and PAOLA1, assessed the efficacy of PARP inhibitors (veliparib, niraparib, olaparib, and olaparib plus bevacizumab, respectively) as first-line maintenance treatment in patients with newly diagnosed advanced ovarian cancer after a response to first-line platinum-based chemotherapy. ICEBERG 3, another 3-arm RCT, evaluated twice-daily continuous olaparib at doses of 200 or 400 mg vs. intravenous infusions of pegylated liposomal doxorubicin (PLD) as a recurrent treatment in advanced ovarian cancer, where two groups with different doses were combined into one group. In the rest of the trials, Kummar et al. (27) and SOLO3 also focused on the PARP inhibitors as recurrent treatment, while ARIEL 3, Study 19, NOVA, SOLO2, and Oza et al. (29) defined the contribution of PARP inhibitors as the second-line or later maintenance treatment in relapsed ovarian cancer patients who had achieved a complete or partial clinical response to their last platinum-based regimen. Most of the included patients with recurrent ovarian cancer were sensitive to their most recent platinum-based chemotherapy, except for a few patients in the trial published by Kaye et al. (26) and Kummar et al. (27). Of these included 12 trials, SOLO1, SOLO2, SOLO3, and ICEBERG 3 only included those patients with mutations of *BRCA*. The risk of bias for each study was assessed according to the Cochrane Handbook 5.1.0 evaluation criteria (Figure 2). The four open-label randomized trials were evaluated as high risk of performance bias.

**Table 1:** Main characteristics of included trials.

**References, study**	**Design**	**No. of Pts (Int/Con)**	**Intervention arm**	**Control arm**	**Timing of treatment**	**Respond to platinum-based therapy**	**No. of *BRCA*m Pts (%)**	**No. of HRD Pts (%)**	**No. of *BRCA*w Pts (%)**	**Median PFS, months (Int/Con)**	**PFS HR (95% CI)**
Coleman et al. (23), ARIEL 3	Phase III, double-blind	564 (375/189)	Rucaparib 600 mg twice daily	Placebo	Second-line or later maintenance treatment	Sensitive	196 (34.8)	354 (62.8)	368 (65.2)	10.8/5.4	0.36 (0.30–0.45)
Coleman et al. (24), VELIA	Phase III, double-blind	1,140 (382/383/375)^a^	Veliparib 150 mg twice daily plus PC followed by veliparib 300/400 mg twice daily maintenance (the veliparib-throughout group)^b^; Veliparib 150 mg twice daily plus PC followed by placebo maintenance (the veliparib-combination-only group)	Placebo plus PC followed by placebo maintenance	First-line maintenance treatment (the veliparib-throughout group); Primary treatment (the veliparib-combination-only group)	NA	298 (26.1)	627 (55.0)	742 (65.1)	22.5/17.3 (the veliparib-throughout group); 15.2/17.3 (the veliparib-combination-only group)	0.68 (0.56–0.83) (the veliparib-throughout group); 1.07 (0.90–1.29) (the veliparib-combination-only group)
González-Martín et al. (25), PRIMA	Phase III, double-blind	733 (487/246)	Niraparib 300 mg once daily^c^	Placebo	First-line maintenance treatment	NA	223 (30.4)	373 (50.9)	399 (54.4)	13.8/8.2	0.62 (0.50–0.76)
Kaye et al. (26), ICEBERG 3	Phase II, open-label	97 (64/33)	Olaparib 200 or 400 mg twice per day	PLD	Recurrent treatment	Sensitive and resistant	97 (100)	97 (100)	0 (0)	6.5/7.1 (200 mg) 8.5/7.1 (400 mg)	0.88 (0.51–1.56)
Kummar et al. (27)	Phase II, open-label	75 (37/38)	Veliparib 60 mg once daily plus oral cyclophosphamide	Oral cyclophosphamide	Recurrent treatment	Sensitive and resistant	31 (41.3)	NA	1 (1.3)	2.1/2.3	NA^d^
Ledermann et al. (14, 21, 22), Study 19	Phase II, double-blind	265 (136/129)	Olaparib 400 mg twice a day (capsules)	Placebo	Second-line or later maintenance treatment	Sensitive	136 (51.3)	NA	118 (44.5)^e^	8.4/4.8	0.35 (0.25–0.49)
Mirza et al. (18), NOVA	Phase III, double-blind	553 (372/181)	Niraparib 300 mg once daily	Placebo	Second-line or later maintenance treatment	Sensitive	250 (45.2)	365 (66.0)	249 (45.0)	NA	0.38 (0.30–0.49)^f^
Moore et al. (28), SOLO1	Phase III, double-blind	391 (260/131)	Olaparib 300 mg twice daily (tablets)	Placebo	First-line maintenance treatment	NA	391 (100)	391 (100)	0 (0)	NA	0.30 (0.23–0.41)
Oza et al. (29)	Phase II, open-label	162 (81/81)	Olaparib 200 mg twice daily plus PC followed by olaparib 400 mg twice daily maintenance (capsules)	PC alone without further treatment	Second-line or later maintenance treatment	Sensitive	41 (25.3)	NA	66 (40.7)	12.2/9.6	0.51 (0.34–0.77)
Penson et al. (30), SOLO3	Phase III, open-label	266 (178/88)	Olaparib 300 mg twice daily (tablets)	Physician's choice single-agent chemotherapy^g^	Recurrent treatment	Sensitive	266 (100)	266 (100)	0 (0)	13.4/9.2	0.62 (0.43–0.91)
Pujade-Lauraine et al. (15), SOLO2	Phase III, double-blind	295 (196/99)	Olaparib 300 mg twice daily (tablets)	Placebo	Second-line or later maintenance treatment	Sensitive	295 (100)	295 (100)	0 (0)	19.1/5.5	0.30 (0.22–0.41)
Ray-Coquard et al. (31), PAOLA1	Phase III, double-blind	806 (537/269)	Olaparib 300 mg twice daily (tablets) plus bevacizumab	Bevacizumab	First-line maintenance treatment^h^	NA	241 (29.9)	387 (48.0)	565 (70.1)	22.1/16.6	0.59 (0.49–0.72)

*No., number; Pts, patients; Int, intervention arm; Con, control arm; BRCAm, BRCA mutated; HRD, homologous-recombination deficiency; BRCAw, BRCA wild-type; PFS, progression-free survival; HR, hazard ratio; CI, confidence intervals; NA, not available; PC, paclitaxel and carboplatin; PLD, pegylated liposomal doxorubicin*.

a *A total of 382 patients was included in the veliparib-throughout group, 383 in the veliparib-combination-only group and 375 in the control group*.

b *After completing chemotherapy, patients received veliparib at a dose of 300 mg twice daily for 2 weeks (transition period) and then veliparib at a dose of 400 mg if the dose in the transition period was not without associated side effects*.

c *Patients received niraparib 200 mg once daily with a baseline body weight of <77 kg, a platelet count of <150,000 per cubic millimeter, or both*.

d *The primary endpoint of this trial was Overall Response Rate (ORR) and the data of PFS was not available*.

e *Wild-type BRCA included patients with no known BRCA mutation and those with a BRCA mutation of unknown significance*.

f *The HR of the whole population from NOVA which was not shown directly was calculated from the subgroups using the generic inverse of variance method*.

g *Physician's choice single-agent chemotherapy contained paclitaxel, topotecan, pegylated liposomal doxorubicin, or gemcitabine*.

h *Before the first-line maintenance treatment, patients were required to have had a clinical complete or partial response to primary treatment with platinum–taxane chemotherapy plus bevacizumab*.

### Both PFS and ORR Were Increased Significantly With the Treatment of PARP Inhibitors, While no OS Advantage Was Observed

**Figure 3:**
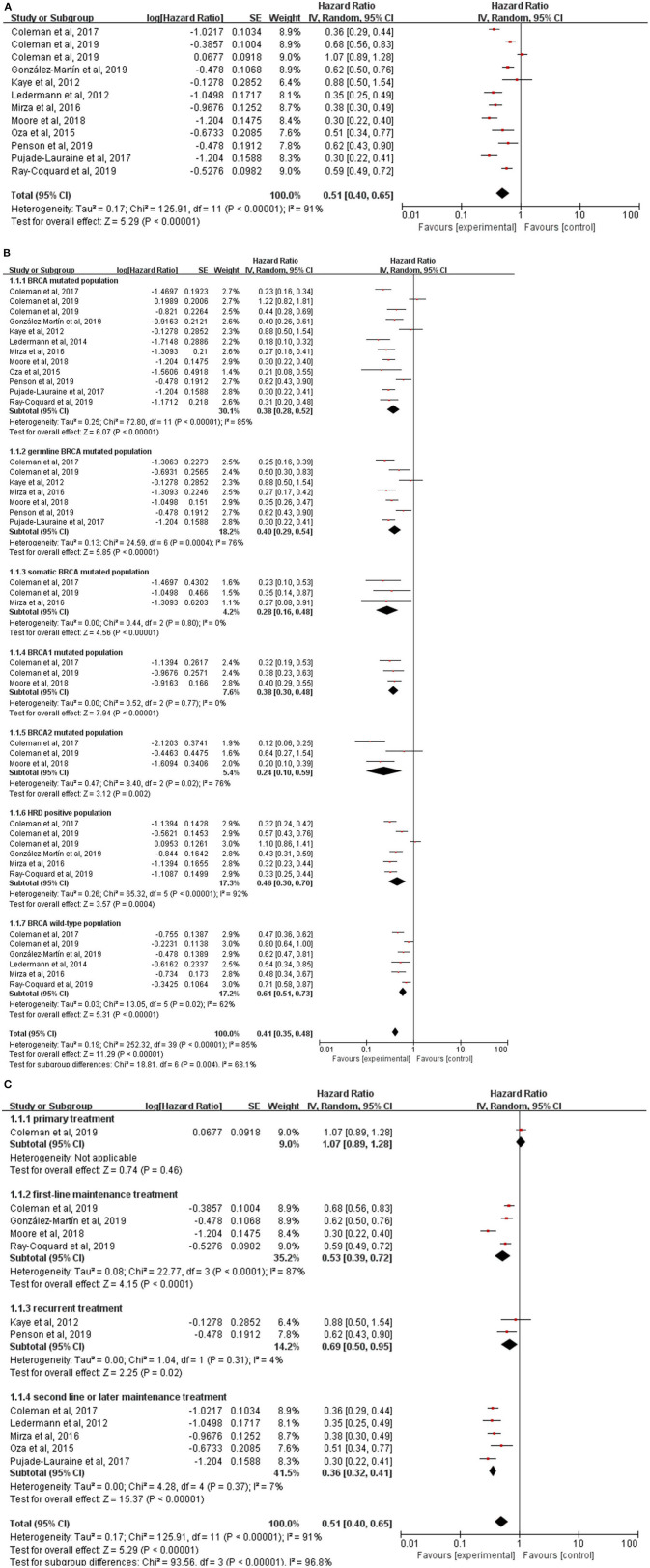
**(A)** Forest plot of hazard ratios (HRs) comparing progression-free survival (PFS) of patients treated with PARP inhibitors vs. placebo or chemotherapy; the HRs plot for PFS of subgroup analysis by genes mutational status **(B)** and treatment lines **(C)**.

The data of PFS from all studies but one (27) were available for meta-analysis. However, the HR of PFS from the NOVA trial, which was not reported directly, was calculated from the data of germline *BRCA* mutation (g*BRCA*) cohort and non-g*BRCA* cohort by the generic inverse of variance method. The overall result showed that PFS (HR, 0.51; 95% CI, 0.40–0.65; *P* < 0.00001) (Figure 3A) was statistically significantly improved with the treatments of PARP inhibitors compared with the treatments of placebo or chemotherapy. Due to a high heterogeneity across the 11 trials (*I*^2^ = 91%; *P* < 0.00001), the random-effect model was used to calculate the pooled HR and two subgroup analyses based on genes mutational status and the timing of treatment were performed.

The population were divided into seven subgroups according to the *BRCA* genes mutations and homologous-recombination status (Figure 3B). Of note, HRs of *BRCA* mutated population in NOVA, g*BRCA* mutated population in SOLO1, HRD population in NOVA, *BRCA* wild-type population in ARIEL 3, PRIMA and NOVA were not obtained directly which were calculated from the HRs in subgroups using the generic inverse of variance method. The result showed that in each subgroup, *BRCA* mutated population (HR, 0.38; 95% CI, 0.28–0.52; *P* < 0.00001), germline *BRCA* mutated population (HR, 0.40; 95% CI, 0.29–0.54; *P* < 0.00001), somatic *BRCA* mutated population (HR, 0.28; 95% CI, 0.16–0.48; *P* < 0.00001), *BRCA1* mutated population (HR, 0.38; 95% CI, 0.30–0.48; *P* < 0.00001), *BRCA2* mutated population (HR, 0.24; 95% CI, 0.10–0.59; *P* = 0.002), HRD positive population (HR, 0.46; 95% CI, 0.30–0.70; *P* < 0.00001) and *BRCA* wild-type population (HR, 0.61; 95% CI, 0.51–0.73; *P* < 0.00001), treatment with a PARP inhibitor compared with placebo or chemotherapy was all significantly associated with an improvement in PFS. It implied that *BRCA* mutation status and homologous-recombination status might contribute to the existing heterogeneity to some extent (*P* = 0.004).

Additionally, we further performed another subgroup analysis stratified by the timing of treatment to provide information for clinicians to choose a suitable time to use PARP inhibitors for patients with ovarian cancer (Figure 3C). As the primary treatment for patients with newly diagnosed advanced ovarian cancer, PC plus veliparib failed to prolong PFS (HR, 1.07; 95% CI, 0.89–1.28; *P* = 0.46) compared with PC plus placebo. There were four trials (VELIA, PRIMA, SOLO1, and PAOLA1) testing the efficacy of PARP inhibitors (veliparib, niraparib, olaparib, and olaparib plus bevacizumab, respectively) as the first-line maintenance therapy and the result revealed a longer PFS (HR, 0.53; 95% CI, 0.39–0.72; *P* < 0.0001) with the treatment of PARP inhibitors. Notably, the PFS was measured from the start of the chemotherapy in VELIA, in contrast to PRIMA, SOLO1, and PAOLA1 of a PARP inhibitor used only as maintenance therapy. The pooled result of ICEBERG 3 and SOLO3 showed olaparib could significantly improve PFS (HR,0.69; 95% CI, 0.50–0.95; *P* = 0.02) as the recurrent treatment for advanced ovarian cancer. Interestingly, the control arm in these two trials was not a placebo but chemotherapy like PLD, paclitaxel, topotecan, and gemcitabine. Kummar et al. also evaluated the efficacy of veliparib plus cyclophosphamide as the recurrent treatment in ovarian cancer but no data of PFS was extracted. In the remaining 5 trials, a significant survival benefit was also investigated in the subgroup of second-line or later maintenance treatment (HR, 0.36; 95% CI, 0.32–0.41; *P* < 0.00001). However, the high heterogeneity (*I*^2^ = 87%, *P* < 0.0001) still existed in the subgroup of first-line maintenance treatment and the random-effect model was employed. Interaction between the timing of treatment and PFS was observed in the test for subgroup differences (*P* < 0.00001).

**Figure 4:**
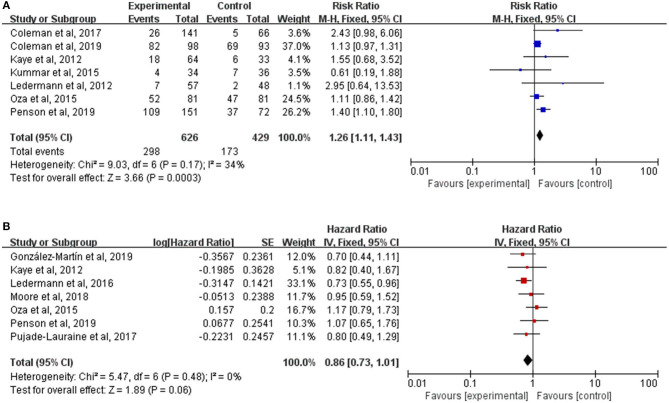
**(A)** Forest plot of risk ratios (RRs) comparing overall response rate (ORR) of patients treated with PARP inhibitors vs. placebo or chemotherapy; **(B)** Forest plot of hazard ratios (HRs) comparing overall survival (OS) of patients treated with PARP inhibitors vs. placebo or chemotherapy.

Not all trials reported data on the secondary endpoints, such as ORR and OS, which were available in seven trials, respectively. There was a significant improvement of ORR for PARP inhibitors therapy (RR, 1.26; 95% CI, 1.11–1.43; *P* = 0.0003) (Figure 4A). But the pooled HR of 0.86 (95% CI, 0.73–1.01; *P* = 0.06) (Figure 4B) failed to show a longer OS in the PARP inhibitors group than the control group by using a fixed-effect model (*I*^2^ = 0%; *P* = 0.48). No interactions between genes mutational status (*P* = 0.64) or the timing of treatment (*P* = 0.77) and OS were observed in subgroup analyses (Supplementary Figure 1).

### PARP Inhibitors Were Associated With a Statistically Significant Higher Risk of Hematologic Events and Different PARP Inhibitors Had Different Toxicities Profiles

**Figure 5:**
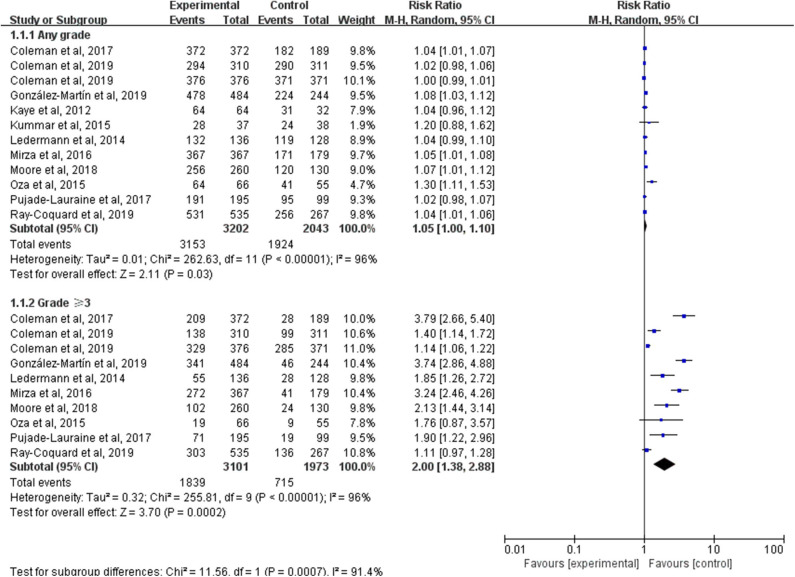
Forest plot of risk ratios (RRs) comparing adverse events (AEs) of any grade or grade 3 or higher in patients treated with PARP inhibitors vs. placebo or chemotherapy.

Except the SOLO3 trial, all studies reported AEs which were graded according to the National Cancer Institute Common Terminology Criteria for Adverse Events (CTCAE), version three or four. Notably, the intervention group in Oza et al. (29) and the veliparib-throughout group in VELIA were PARP inhibitors plus chemotherapy followed by PARP inhibitors alone maintenance and only the AEs at the monotherapy maintenance phase were analyzed. But AEs during the whole treatment phase (both combination phase and maintenance phase) were assessed between the veliparib-combination-only group and the control group in VELIA. By pooling the results of 11 trials, AEs of any grade occurred in 3,153 of 3,202 (98.47%) patients in the PARP inhibitors group and 1,634 of 1,732 (94.34%) patients in the control group (RR, 1.05; 95% CI, 1.00–1.10; *P* = 0.03) (Figure 5). To avoid double-counting, the number of people with AEs during the monotherapy maintenance phase in the control arm of VELIA was not counted in the total control group. However, the meta-analysis of 10 trials with available data showed that patients treated with a PARP inhibitor were at a higher risk of grade 3 or worse AEs than those treated with placebo or chemotherapy (RR, 2.00; 95% CI, 1.38–2.88; *P* = 0.0002). Of note, the above two results were both calculated by a random-effect model because of the high heterogeneity (*I*^2^ = 96%, *P* < 0.00001). To further explore the potential sources of heterogeneity, we conducted two subgroup analyses according to the type of AEs and the type of PARP inhibitors.

The result of our subgroup analysis by the type of AEs is showed in Table 2. Anemia was the most common sever AEs which was reported in 697 of 3,202 patients in the PARP inhibitors therapy group and 109 of 1,732 in the placebo or chemotherapy group (RR, 10.96; 95% CI, 3.14–38.23; *P* = 0.0002). Patients treated with PARP inhibitors were also at a higher risk for another two hematologic events, thrombocytopenia (RR,7.38; 95% CI, 2.10–25.88; *P* = 0.002) and neutropenia (RR, 2.91; 95% CI, 1.55–5.44; *P* = 0.0008). Additionally, statistically higher incidences of fatigue (RR, 2.57; 95% CI, 1.64–4.03; *P* < 0.0001) and nausea (RR, 2.33; 95% CI, 1.46–3.72; *P* = 0.0004) were also observed in the PARP inhibitors treatment group. However, differences in RRs for vomiting (*P* = 0.07), abdominal pain (*P* = 0.73), diarrhea (*P* = 0.97), and long-term hematologic events (*P* = 0.36) between the PARP inhibitors therapy group and the control group may not be meaningful. Heterogeneity was still high in treatment-emergent hematologic toxicities but eliminated in the other six subgroups (*I*^2^ = 26%, *P* = 0.21 fatigue; *I*^2^ = 0%, *P* = 0.68 nausea; *I*^2^ = 0%, *P* = 0.76 vomiting; *I*^2^ = 0%, *P* = 0.63 abdominal pain; *I*^2^ = 0%, *P* = 0.51 diarrhea; *I*^2^ = 0%, *P* = 0.91 long-term hematologic events).

**Table 2:** RRs of grade 3 or higher AEs comparing PARP inhibitors group vs. the control group.

**Adverse event type**	**No. of included trials**	**PARP inhibitors therapy**	**Placebo or chemotherapy**	**RR (95% CI)**	***P***	***I*^**2**^(%)**	***P***
Anemia	11	697/3,202	109/1,732	10.96 (3.14–38.23)	0.0002	91	<0.00001
Thrombocytopenia	9	432/3,002	36/1,572	7.38 (2.10–25.88)	0.002	79	<0.00001
Neutropenia	10	481/3,138	210/1,700	2.91 (1.55–5.44)	0.0008	80	<0.00001
Fatigue	11	162/3,202	34/1,732	2.57 (1.64–4.03)	<0.0001	26	0.21
Nausea	11	89/3,202	19/1,732	2.33 (1.46–3.72)	0.0004	0	0.68
Vomiting	11	63/3,202	23/1,732	1.55 (0.96–2.50)	0.07	0	0.76
Abdominal pain	11	64/3,202	34/1,732	1.08 (0.71–1.63)	0.73	0	0.63
Diarrhea	10	40/2,718	23/1,488	0.99 (0.58–1.67)	0.97	0	0.51
Long-term hematologic events^a^	9	27/3,167	8/1,639	1.40 (0.68–2.87)	0.36	0	0.91

*RRs, risk ratios; AEs, adverse events; No., number*.

a *Long-term hematologic events referred to myelodysplastic syndrome, acute myeloid leukemia, chronic myelomonocytic leukemia, and aplastic anemia*.

A total of 4 PARP inhibitors (rucaparib, veliparib, niraparib, and olaparib) were tested in the included trials and the subgroup analysis showed there was a significant interaction between drug type and AEs. Table 3 presents RRs of any type AEs and three common hematologic toxicities (grade 3 or higher) between the PARP inhibitors group and the control group. Rucaparib, veliparib, and niraparib were all associated with the increased risks of AEs of any type and three common hematologic toxicities (grade 3 or higher). Concerning olaparib, a higher risk of total grade 3 or greater AEs (RR, 1.40; 95% CI, 1.24–1.58), anemia (RR, 15.00; 95% CI, 7.18–31.34), and neutropenia (RR, 1.96; 95% CI, 1.20–3.22) were observed. However, RR of thrombocytopenia of 1.63 (95% CI, 0.53–5.04) was not significant.

**Table 3:** RRs of grade 3 or higher AEs according to drug type.

**Drug type**	**Rucaparib**	**Veliparib**	**Niraparib**	**Olaparib**	***P*^a^**
	**RR (95% CI)**	**RR (95% CI)**	**RR (95% CI)**	**RR (95% CI)**	
Any type	3.79 (2.66–5.40)	1.21 (1.12–1.30)	3.50 (2.89–4.24)	1.40 (1.24–1.58)	<0.00001
Anemia	35.56 (4.98–254.04)	1.76 (1.43–2.16)	27.04 (10.71–68.26)	15.00 (7.18–31.34)	<0.00001
Thrombocytopenia	20.91 (1.26–348.18)	5.70 (3.76–8.64)	94.48 (23.35–382.34)	**1.63 (0.53–5.04)**^**b**^	0.0001
Neutropenia	6.74 (1.58–28.75)	1.63 (1.25–2.14)	13.02 (5.69–29.76)	1.96 (1.20–3.22)	<0.0001

*RRs, risk ratios; AEs, adverse events*.

a *Difference in the RR of different PARP inhibitors*.

b *RR of thrombocytopenia caused by Olaparib treatment was not significant*.

### Sensitivity Analysis

Sensitivity analysis was performed to evaluate the contributions of each study to the pooled results by omitting all the trials one by one. The exclusion of any single trial did not significantly change the overall results of the pooled HRs or RRs (Supplementary Figure 2), indicating our analysis was robust and stable.

### Publication Bias

We performed Begg's funnel plot and Egger's test to detect the publication bias (Supplementary Figure 3). The results showed no obvious publication bias for HRs of PFS or OS and RRs of ORR or AEs (*P* = 0.29 for PFS, *P* = 0.523 for OS, *P* = 0.317 for ORR, *P* = 0.065 for AEs).

## Discussion

In recent years, clinical trials on the therapeutic effect of PARP inhibitors on advanced-stage epithelial ovarian cancer have been widely carried out (32). By pooling the data of 12 RCTs, this updated meta-analysis further our understanding of the efficacy and safety of PARP inhibitors in EOC.

The result demonstrated that PARP inhibitors were statistically significantly associated with a prolonged PFS (pooled HR, 0.51; *P* < 0.00001) compared with placebo or chemotherapy alone. ORR was also improved significantly with an RR of 1.26 (*P* = 0.0003). However, no OS advantage was observed (pooled HR, 0.86; *P* = 0.06). Compared with the former meta-analysis, a total of 12 RCTs with 5,347 patients were analyzed to evaluate the efficacy of PARP inhibitors in terms of PFS, OS, and ORR in our study, which made the conclusions more reliable and persuasive. Among these included trials, 10 set the PFS as the primary endpoint and 2 [Kummar et al. (27) and the SOLO3] set the ORR as the primary endpoint. Interestingly, the pooled HR for OS significantly changed when we excluded the trial published by Oza et al. (29) and the SOLO3 trial. With the extension of the follow-up in Study 19, especially after 36 months, the Kaplan-Meier curve in the overall population and women with *BRCA* mutation began to show benefits in OS with the treatment of olaparib (14, 21, 22). As such, further investigations with long-term follow-up are needed to evaluate the OS advantages and support the PFS advantages of PARP inhibitors therapy in ovarian cancer.

Considering the high heterogeneity in pooling the HR for PFS, two subgroups were performed to explore the potential factors. Historically, PARP inhibitors are subjected to be effective for patients with homologous recombination deficiency (HRD) (33). However, our result of subgroup analysis according to gene mutation status demonstrated that PARP inhibitors could significantly improve the PFS regardless of the presence or absence of *BRCA* mutations or HRD status, although the magnitude of benefit appeared higher in patients with *BRCA*-mutated tumors. A significant PFS advantage with a pooled HR of 0.61(*P* < 0.00001) in wild-type *BRCA* population indicates that PARP inhibitors may have other anti-cancer mechanisms. Recent studies have shown that PARP inhibitors can generate cytosolic dsDNA, which in turn activates the DNA-sensing cGAS-STING pathway and induce IFN-mediated anti-cancer immune responses independent of BRCAness (34). Another two studies also confirmed that PARP inhibitors can involve in anti-cancer immunity (35, 36). Additionally, other complementary mechanisms, such as PARP-regulated rDNA transcription and ribosome biogenesis have also been reported (37). Given the recent data showing different sensibility to therapy between *BRCA* 1 and 2 mutated patients (38, 39), a subgroup analysis of the two subpopulations was performed and the result demonstrated no difference in their response to PARP inhibitors (*I*^2^ = 0%, *P* = 0.34). Interestingly, subgroup analysis between germline and somatic *BRCA* mutated patients also showed no difference in their response to PARP inhibitors (*I*^2^ = 22.8%, *P* = 0.26). In our second subgroup analysis according to the timing of treatment, patients with ovarian cancer were divided into newly treated patients and recurrent patients. The result revealed that there was a significant interaction between the timing of treatment and PFS (*P* < 0.00001). Specifically, pooled results showed substantial PFS benefits with the PARP inhibitors group vs. the control group as first-line maintenance treatment, recurrent treatment, and second-line or later maintenance treatment, while no difference of PFS between the two groups was observed when PARP inhibitors for primary treatment. But there was a non-negligible limitation of this conclusion, that is, the data on the efficacy of PARP inhibitors as primary treatment was totally obtained from VELIA. The limited data are not sufficient to conclude that PARP inhibitors cannot achieve a clinical benefit on their use as primary treatment. Additionally, only veliparib and olaparib vs. chemotherapy were assessed as recurrent treatment. More studies on PARP inhibitors as primary treatment and recurrent treatment in ovarian cancer are needed.

The overall adverse events of PARP inhibitors did not change significantly, but grade 3 or higher adverse events did increase compared with the control group. PARP inhibitors were associated with a statistically significant higher risk of hematologic events (anemia, thrombocytopenia, and neutropenia), fatigue, and nausea. As the reported dose modification and interruptions caused by those adverse events, regular hematological monitoring was recommended (40). Due to the high heterogeneity in terms of the combined RRs of total AEs and hematologic events (*I*^2^ > 50%, *P* < 0.00001), we performed a subgroup analysis according to the drug type and found that there was a significant interaction between drug type and AEs (*P* < 0.0001).

This meta-analysis has many strengths. First, a comprehensive review was performed and data were obtained from 12 randomized controlled trials of 5,347 patients. The quality of these clinical trials and a large number of patients made our conclusions more reliable and persuasive. And more notably, detailed subgroup analyses in this meta-analysis provided clinicians with more information, such as the best time and suitable population to use PARP inhibitors. Unfortunately, the main limitation of our study is the heterogeneity of the study population. Our analysis is based on published results rather than individual patients' data, which made the confounding factors, such as age, FIGO stage, and number of previous platinum-based regimens on the patient level not be controlled.

In future investigations, it might be useful to compare the efficacy and toxicity of antiangiogenic agents and PARP inhibitors in EOC, as they are the most promising strategies among the many targeted therapies currently under evaluation. Additionally, it might be of particular interest to analyze time to second progression in patients treated with PARP inhibitors vs. chemotherapy or placebo, to further explore whether PARP inhibitors diminish patients' ability to benefit from subsequent therapy. And the combinations of PARP inhibitors and other anti-cancer therapies have become increasingly popular to achieve chemo-free therapy (41–43). However, adverse events, especially hematologic toxic effects, will be a primary point of concern and the potential rationale need to be studied.

In conclusion, our results confirmed that PARP inhibitors are an effective and well-tolerated therapy for patients with advanced-stage epithelial ovarian cancer. PARP inhibitors showed encouraging survival benefits in terms of PFS and ORR. It could statistically significantly improve PFS regardless of *BRCA* genes mutations, homologous-recombination status, and treatment lines. However, no difference of OS between the PARP inhibitors group and the control group was observed and further studies should be performed. PARP inhibitors were associated with a statistically significant higher risk of hematologic events and regular examination was recommended. However, different PARP inhibitors may have different toxicities profile.

## Data Availability Statement

All datasets presented in this study are included in the article/Supplementary Material.

## Author Contributions

XY had full access to all the data in the study and take responsibility for the integrity of the data and the accuracy of the data analysis. XY and YJ conceived, designed this research, and wrote the paper. YJ, JZ, LZ, and ST searched the database. JZ, TY, LW, and MZ extracted the data. YJ, QY, and YW analyzed the data and completed the figures and tables. All authors contributed to the article and approved the submitted version.

## Conflict of Interest

The authors declare that the research was conducted in the absence of any commercial or financial relationships that could be construed as a potential conflict of interest.
