# Supplementary material for: Evaluation of the Efficacy and Safety of PARP Inhibitors in Advanced-Stage Epithelial Ovarian Cancer
Source: Front Oncol. 2020 Jul 3;10:954. doi: 10.3389/fonc.2020.00954 (PMC7350528; doi:10.3389/fonc.2020.00954)
Supplement: Supplementary file 1 [file Data_Sheet_1.docx]

(A)


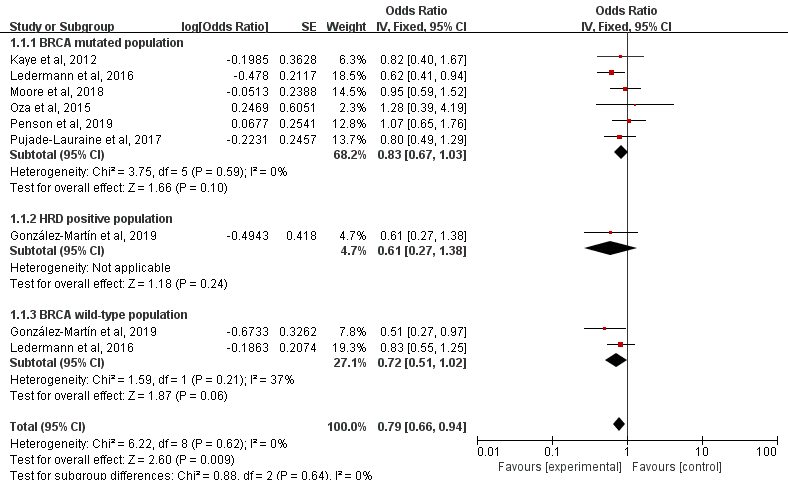


(B)


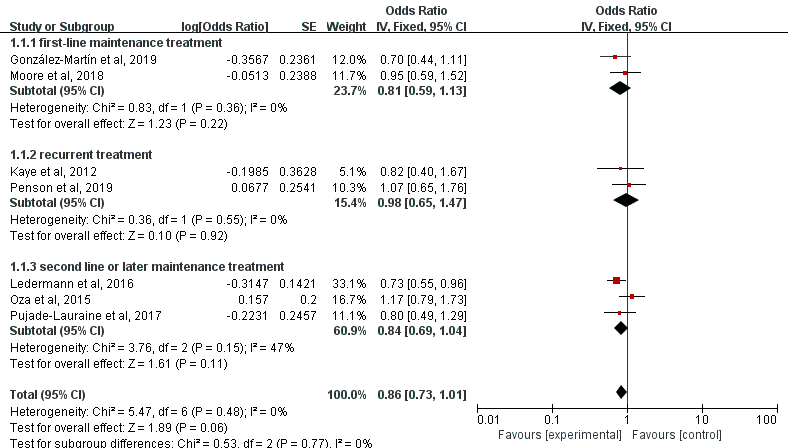


**Supplementary Figure 1.** The hazard ratios (HRs) plot for overall survival (OS) of subgroup analysis by genes mutational status (A) and treatment lines (B).

(A)


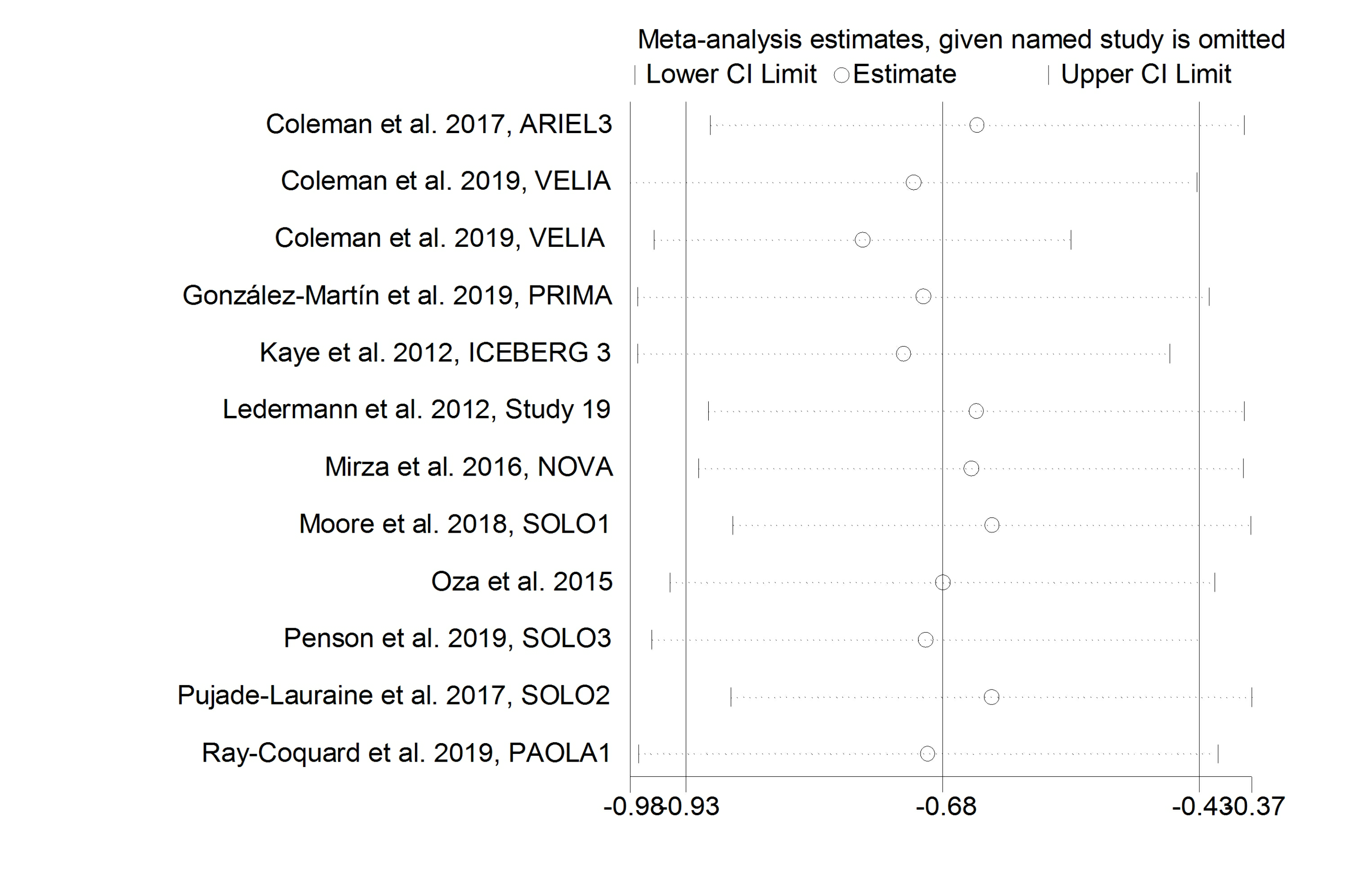


(B)


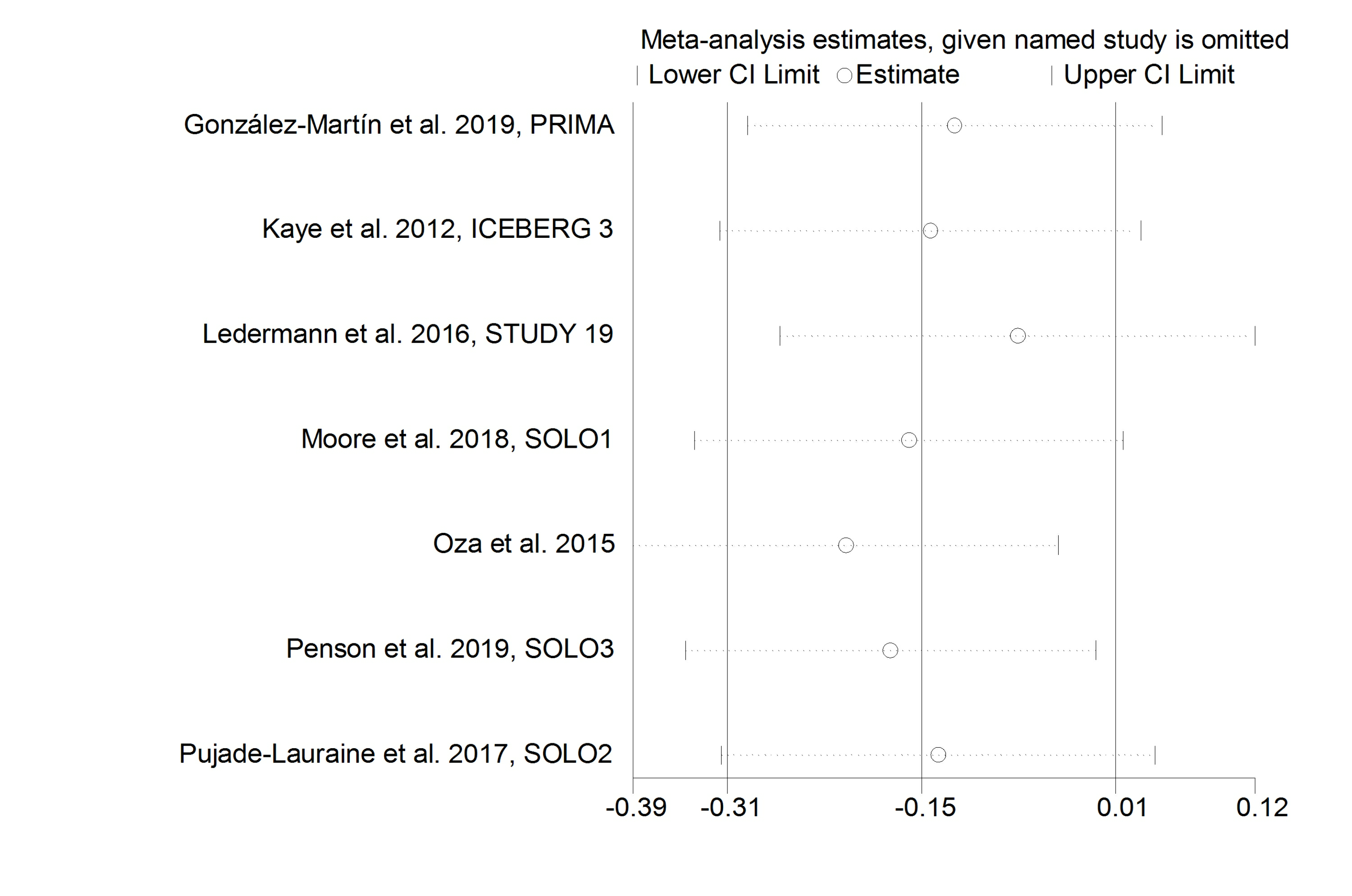


(C)


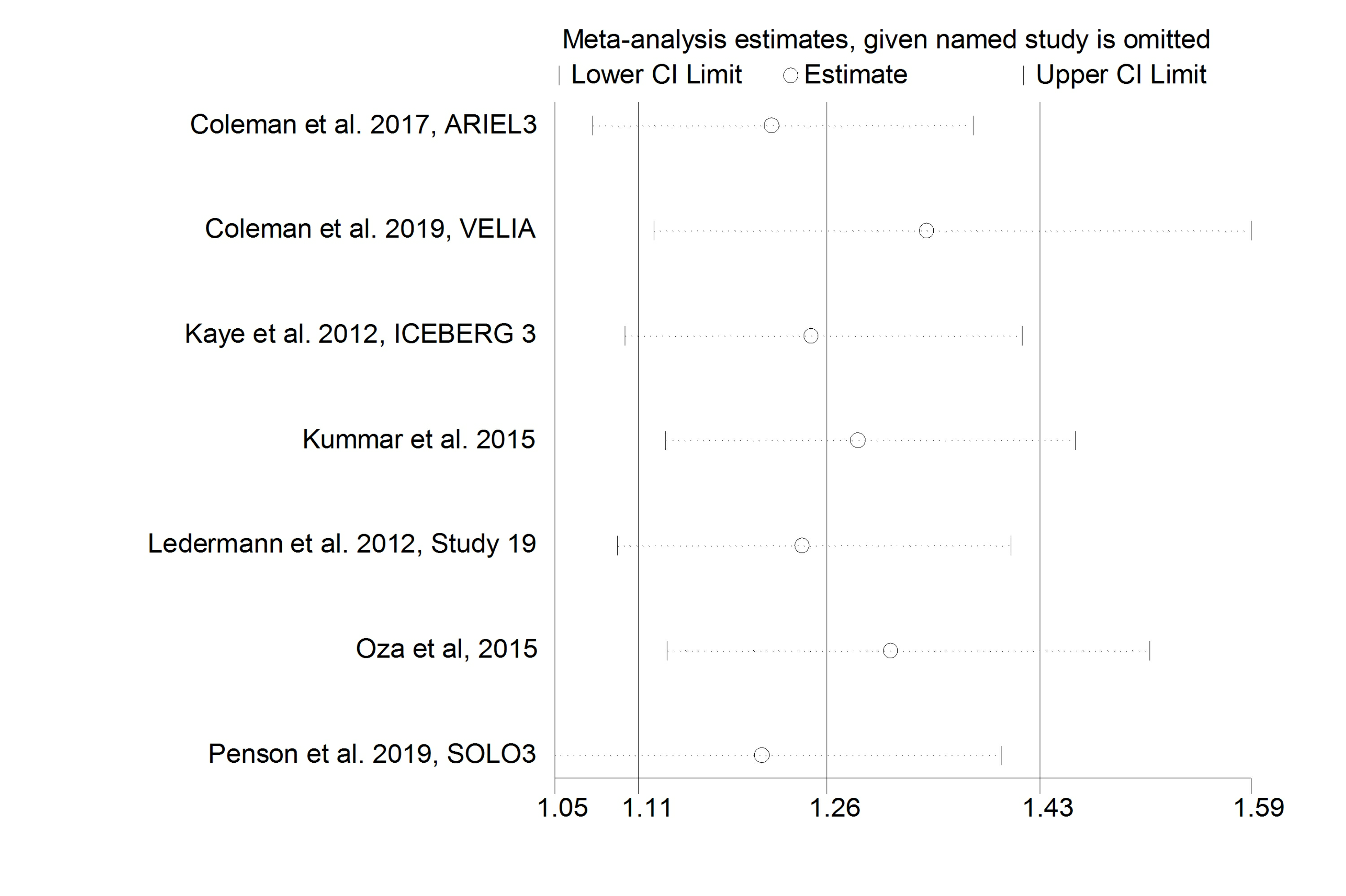


(D)


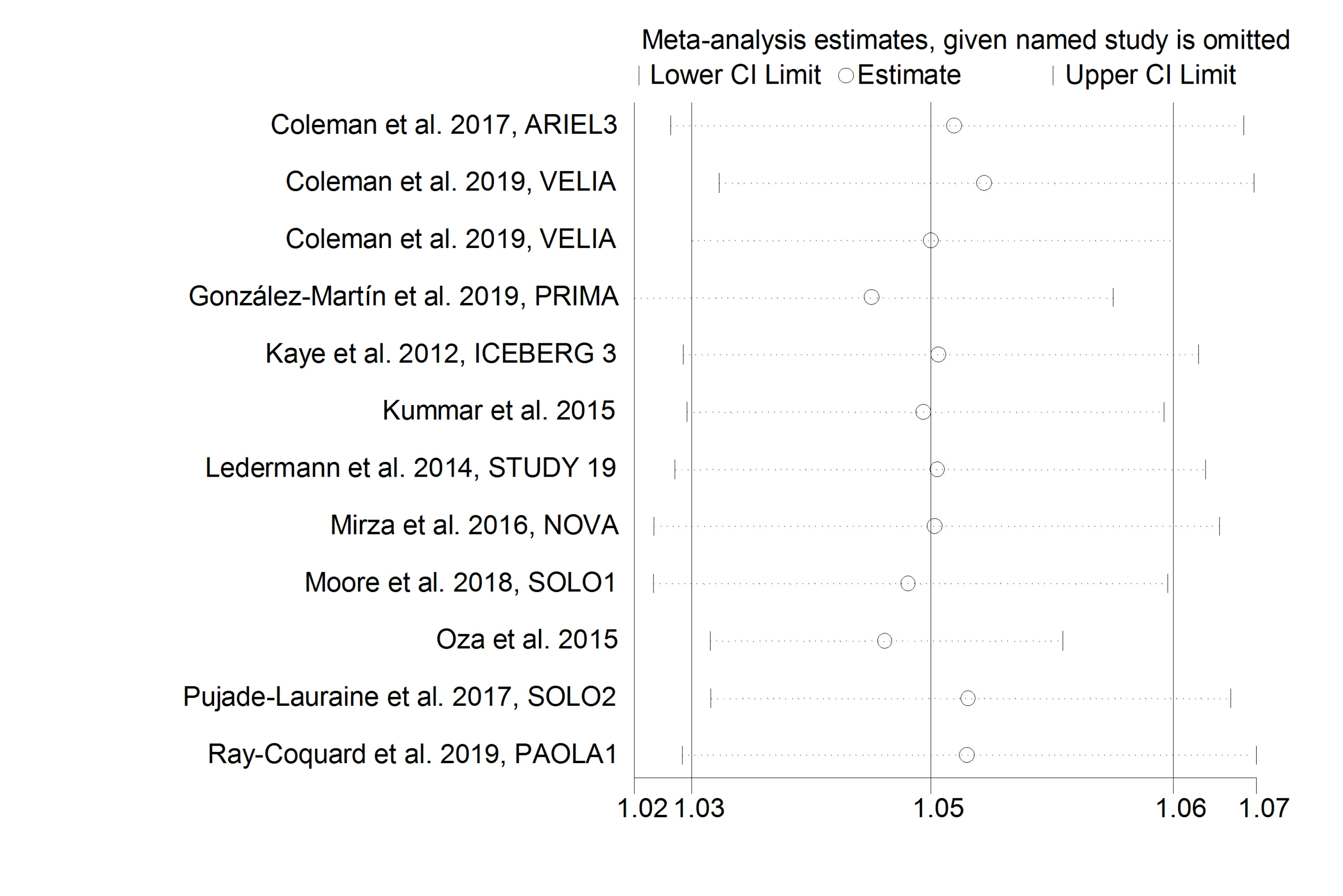


**Supplementary Figure 2.** Sensitivity analysis of the efficacy and safety of PARP inhibitor in the maintenance treatment of advanced stage epithelial ovarian cancer. (A) PFS, (B) OS, (C) ORR, (D) AEs.

(A)

**
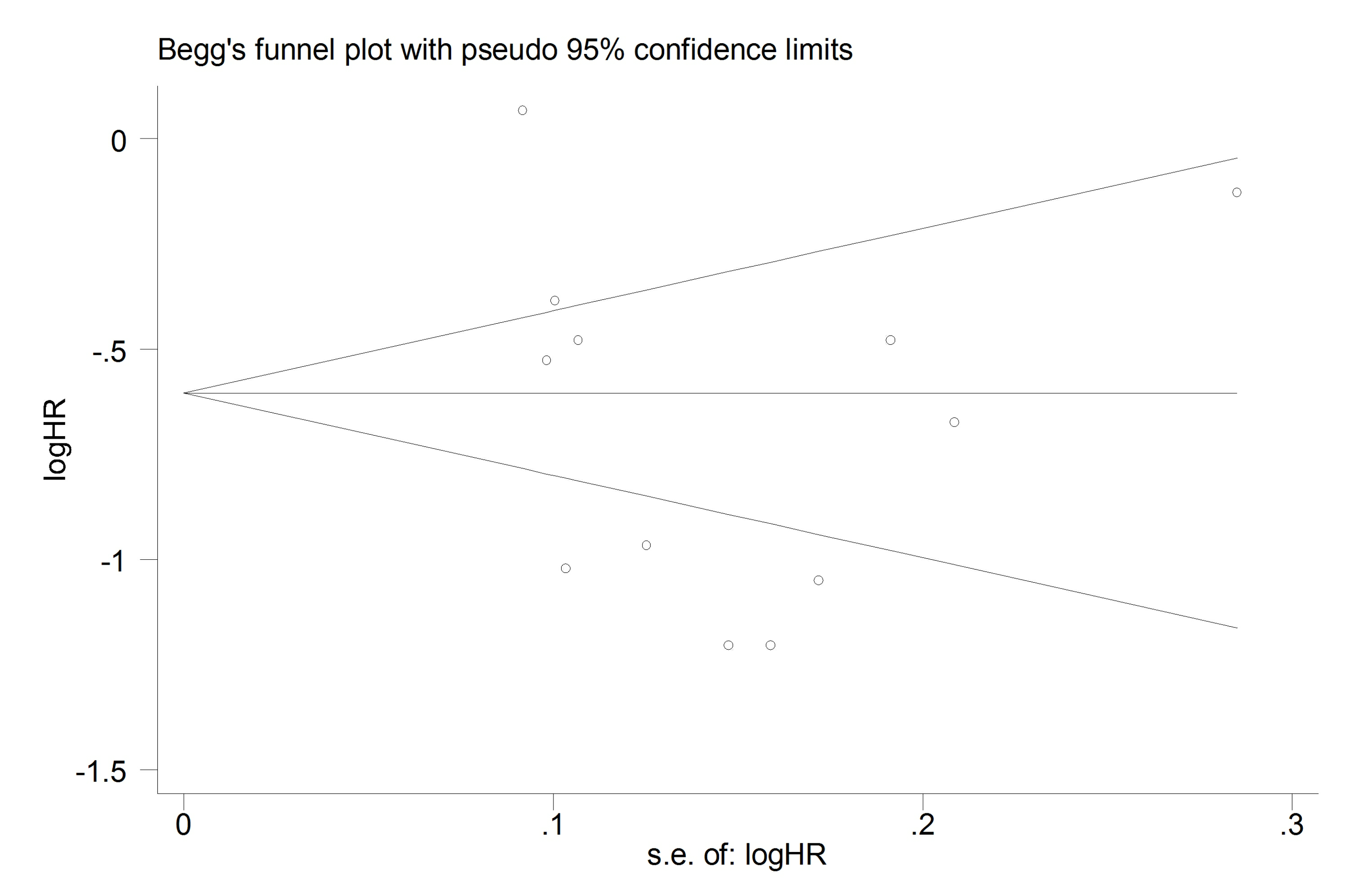
**

(B)


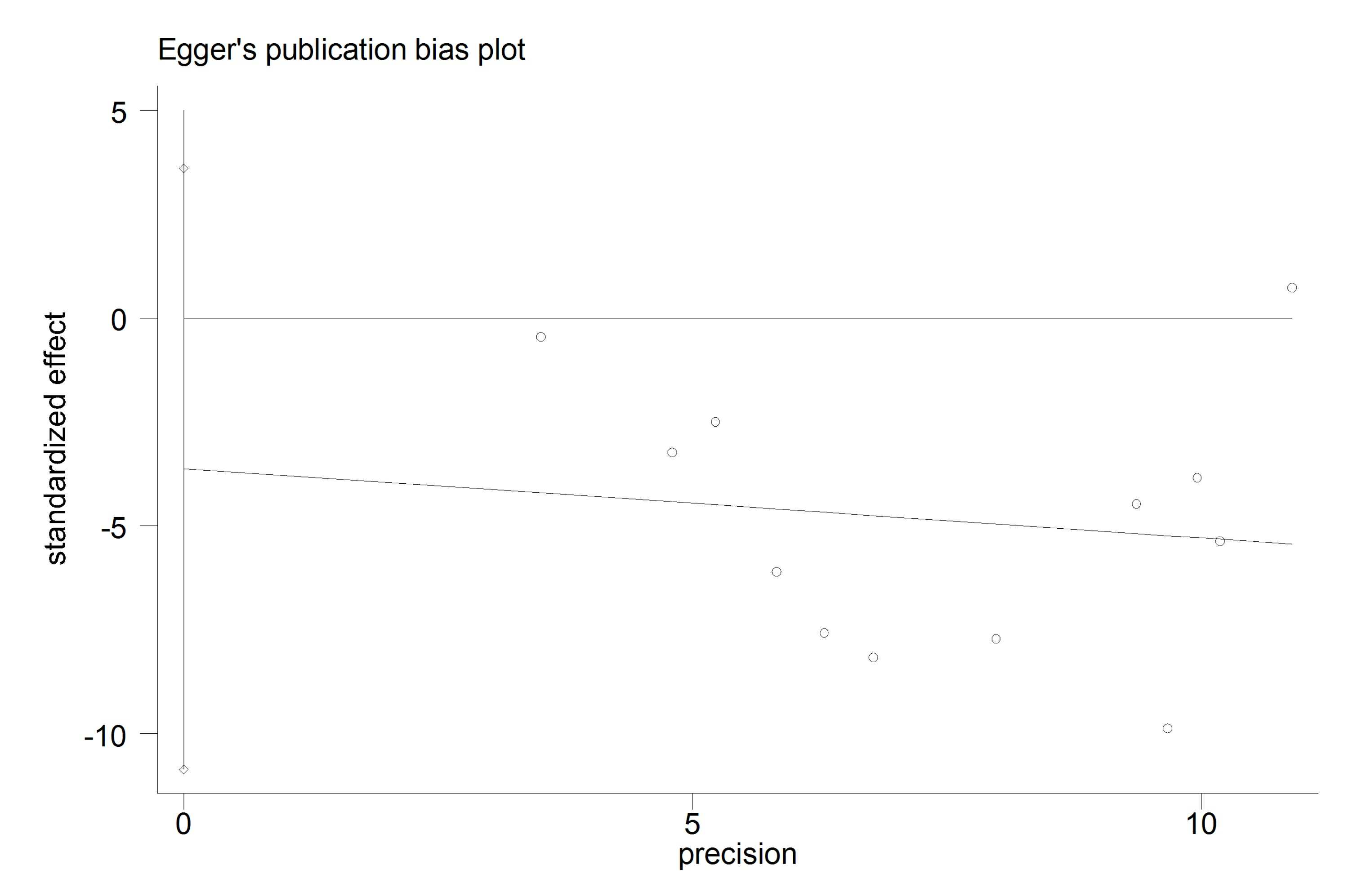


(C)


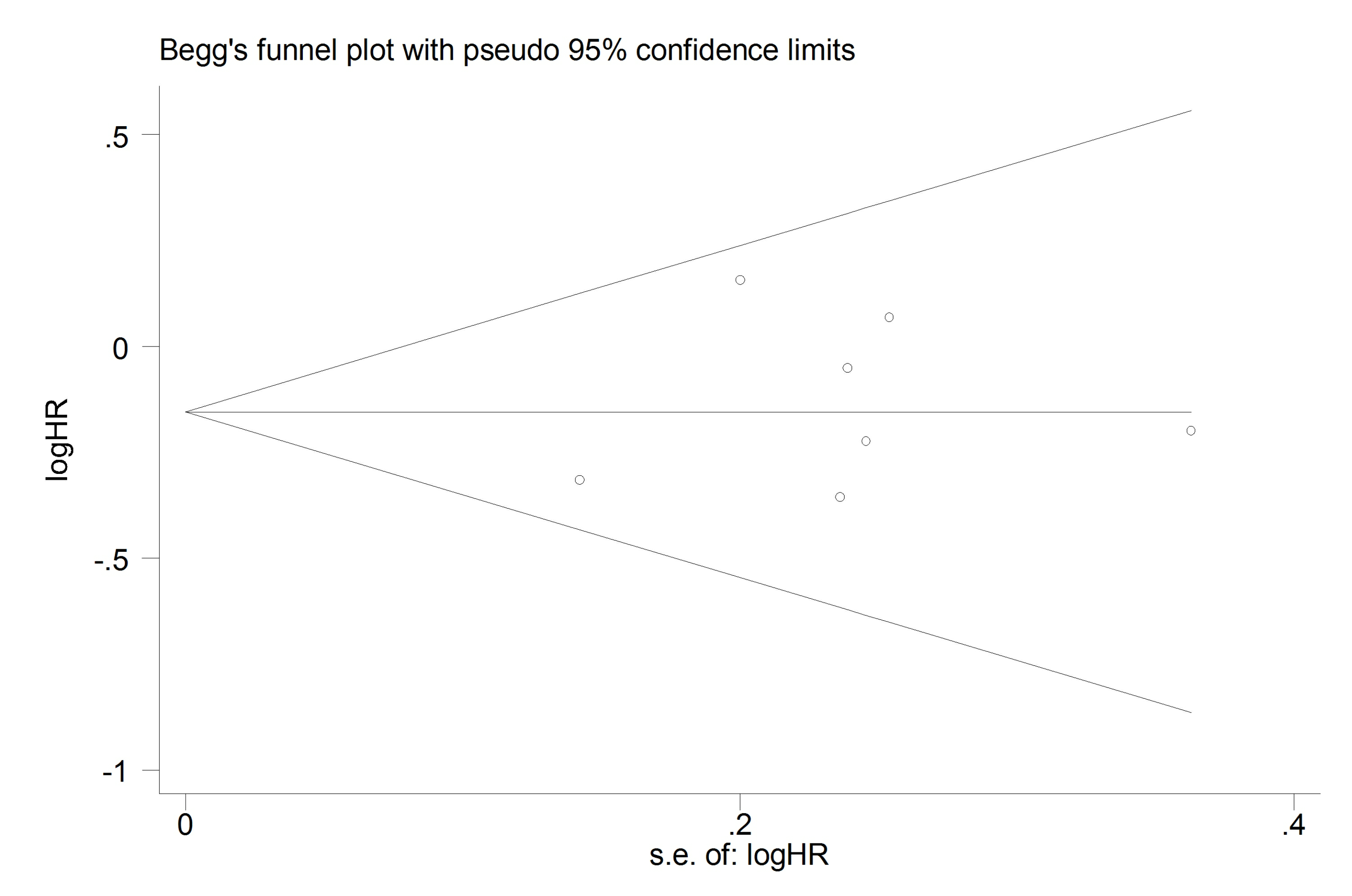


(D)


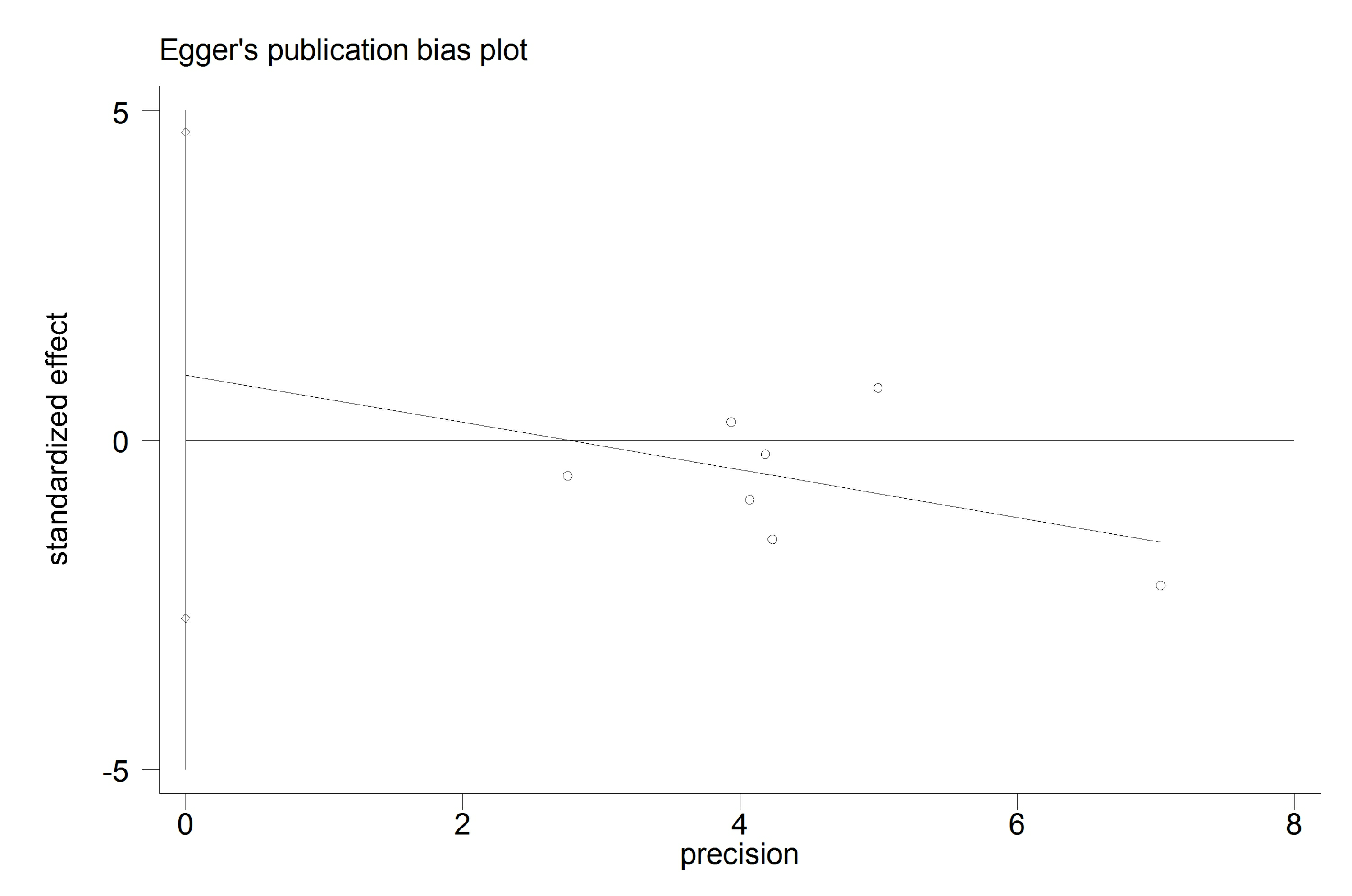


(E)


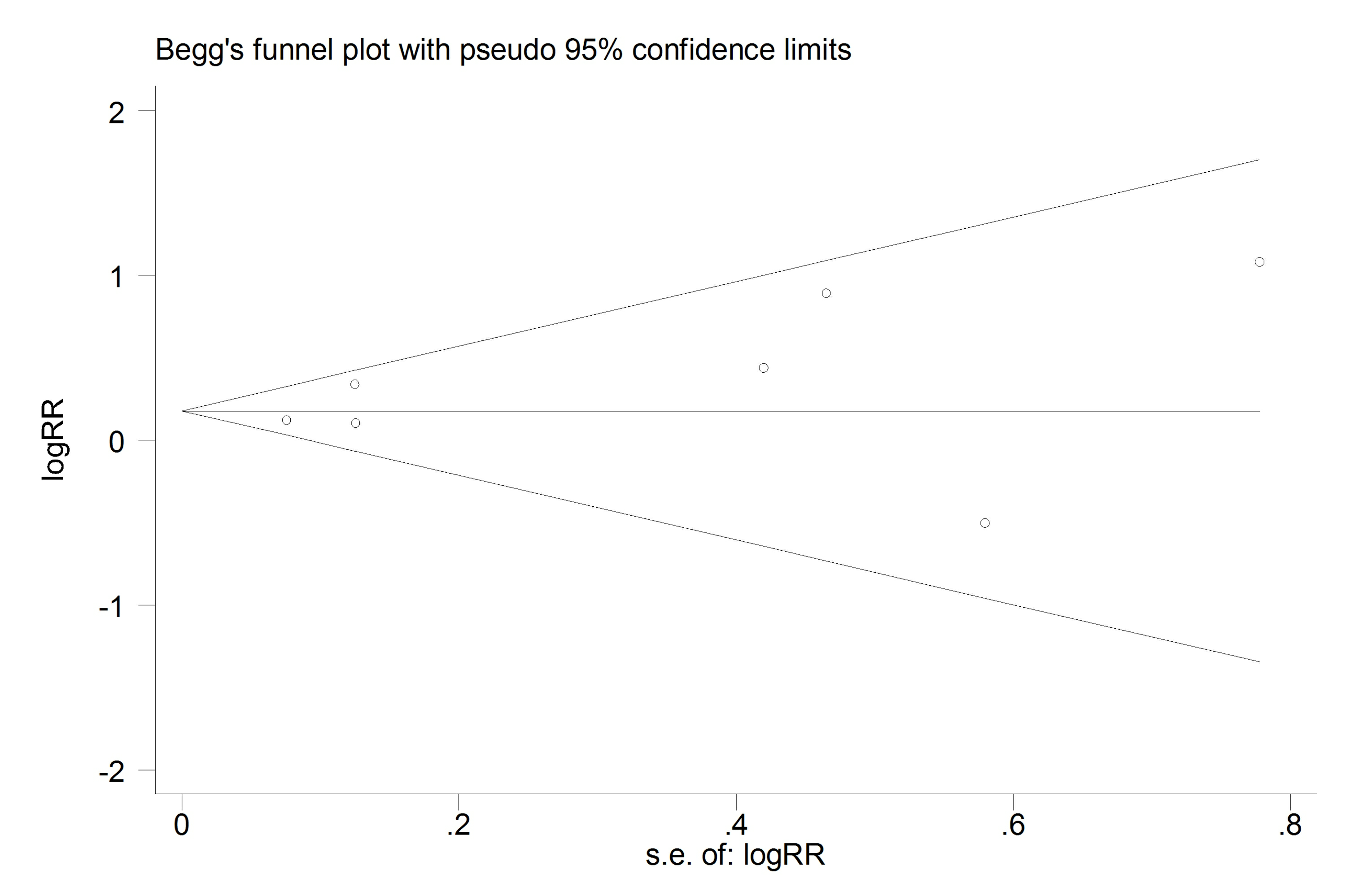


(F)


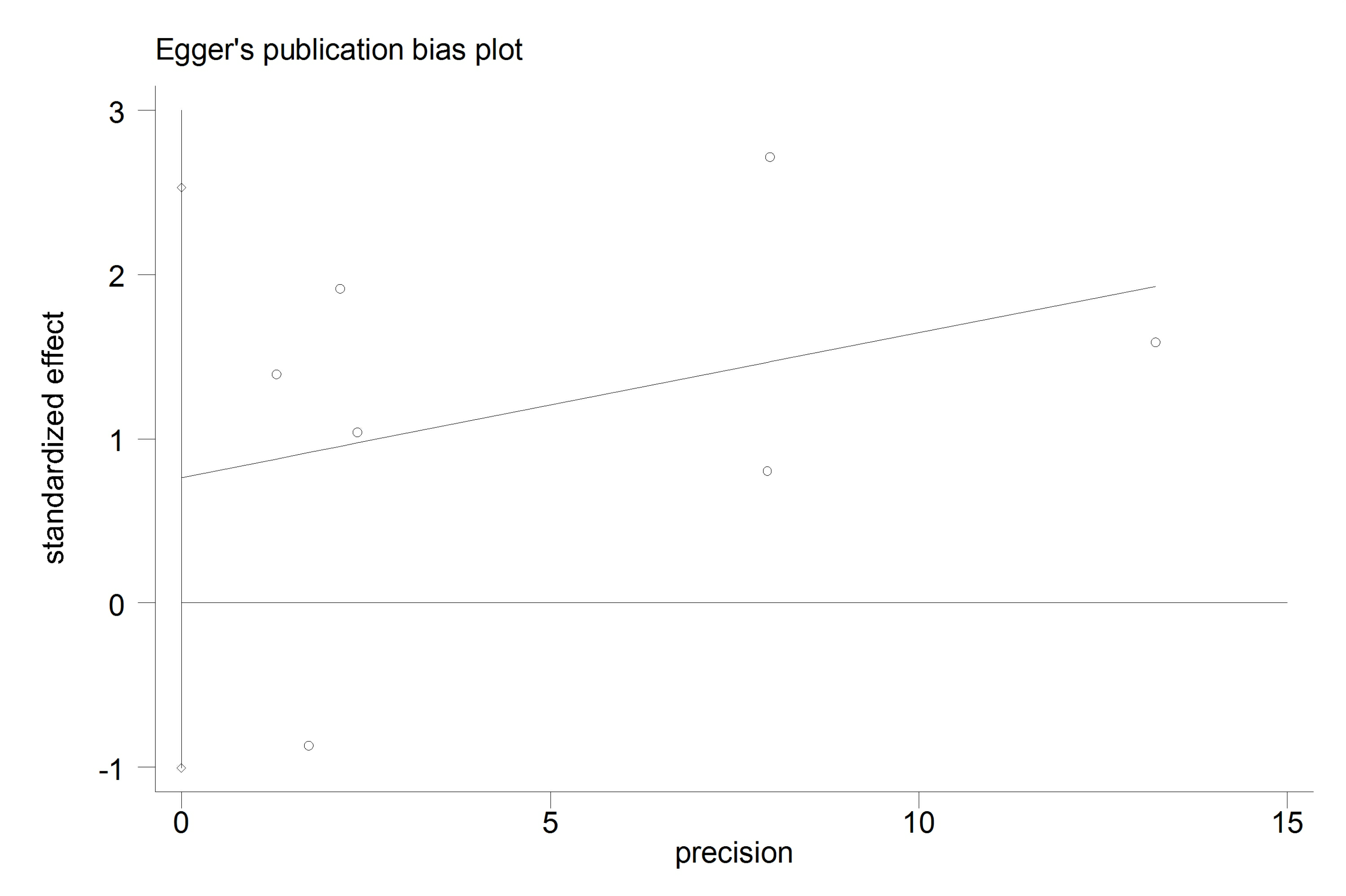


(G)


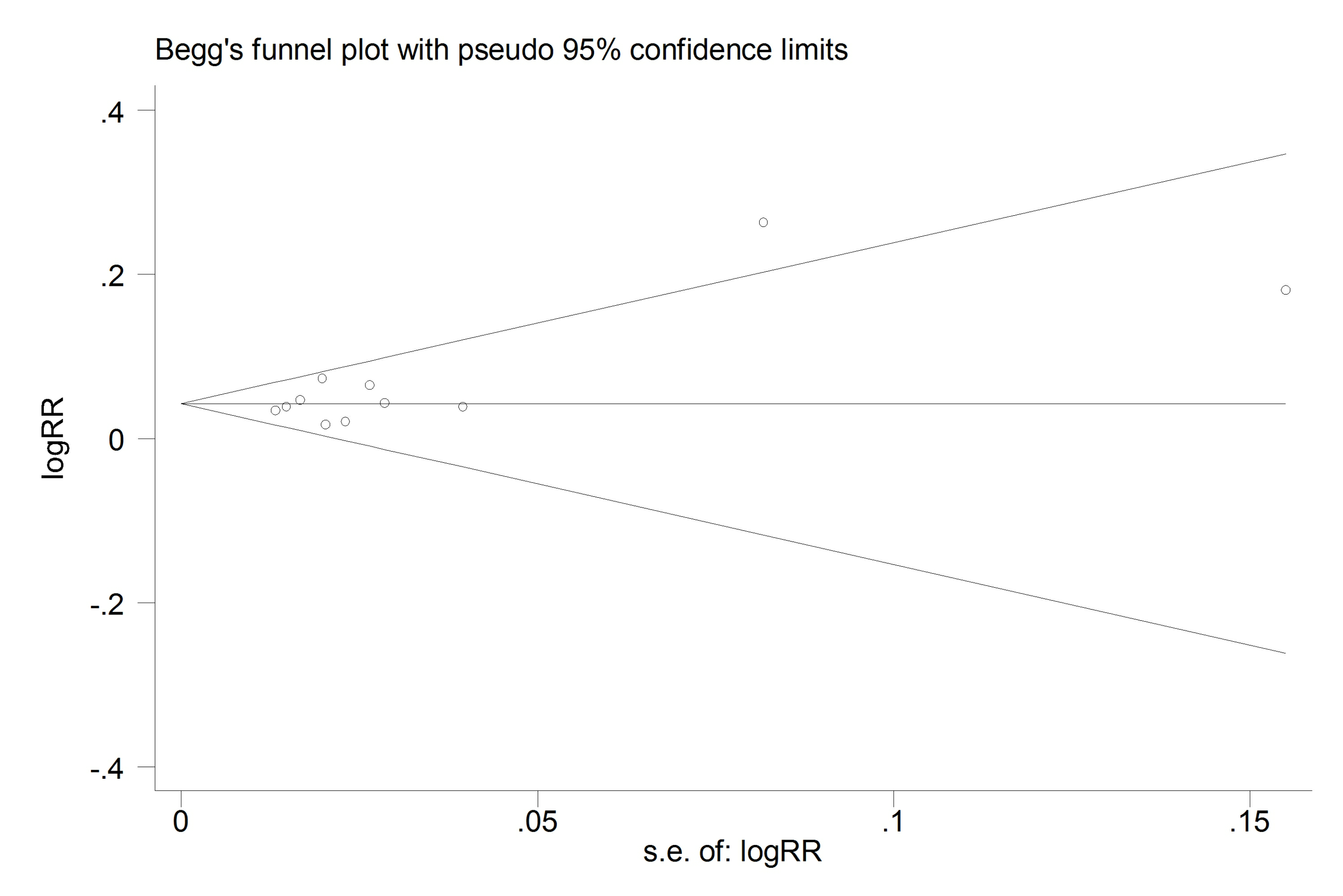


(H)


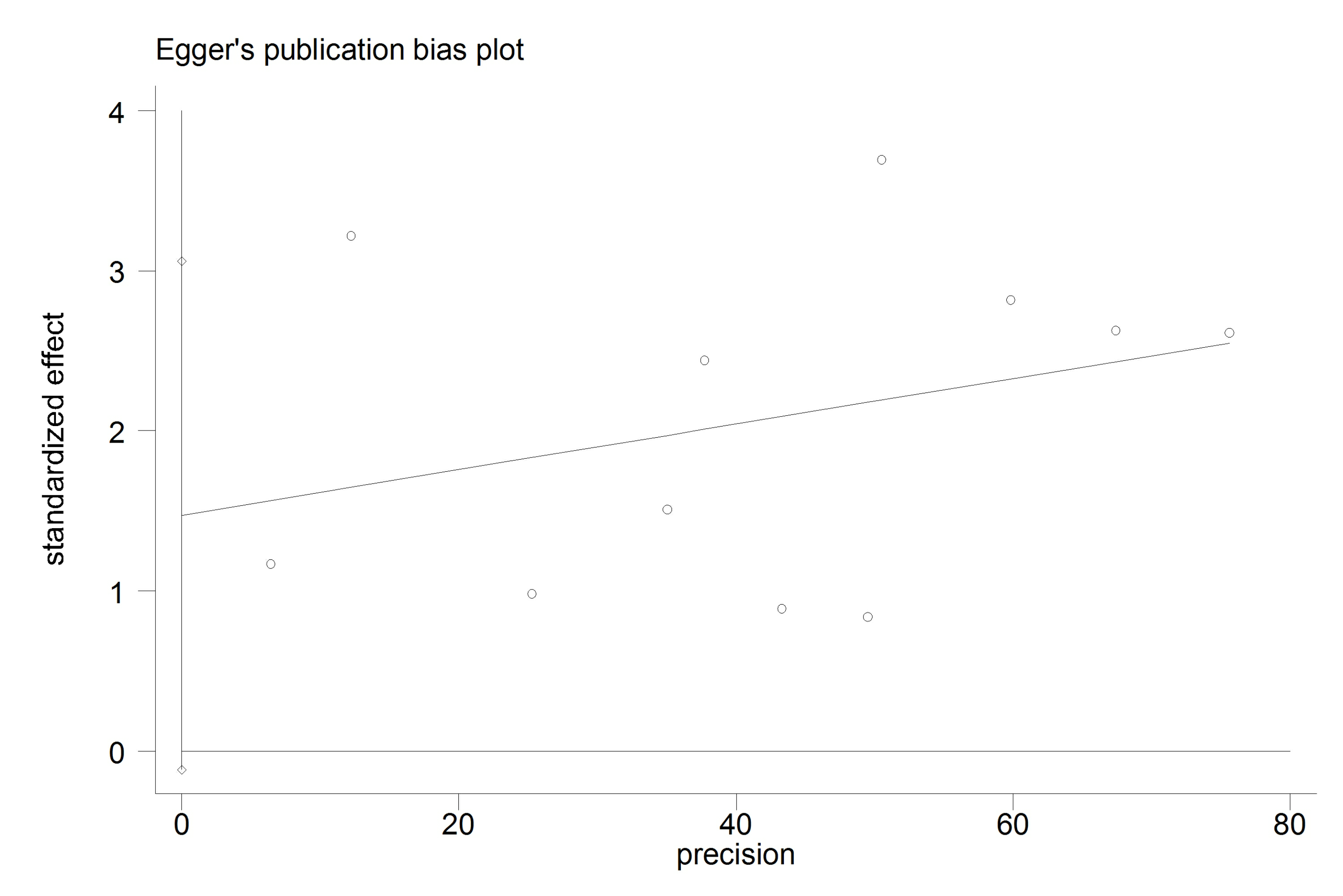


**Supplementary Figure 3.** Publication bias of the efficacy and safety of PARP inhibitor in the maintenance treatment of advanced stage epithelial ovarian cancer. (A, B) PFS (Egger’s test: *P* = 0.290); (C, D) OS (Egger’s test: *P* = 0.523); (E, F) ORR (Egger’s test: *P* = 0.317); (G, H) AEs (Egger’s test: *P* = 0.065).
